# Age and Gender Differences in the Social Patterning of Cardiovascular Risk Factors in Switzerland: The CoLaus Study

**DOI:** 10.1371/journal.pone.0049443

**Published:** 2012-11-13

**Authors:** Silvia Stringhini, Brenda Spencer, Pedro Marques-Vidal, Gerard Waeber, Peter Vollenweider, Fred Paccaud, Pascal Bovet

**Affiliations:** 1 Institute of Social and Preventive Medicine (IUMSP), Lausanne University Hospital, Lausanne, Switzerland; 2 Department of Medicine, Internal Medicine, CHUV and Faculty of Biology and Medicine, Lausanne, Switzerland; Harvard School of Public Health, United States of America

## Abstract

**Objectives:**

We examined the social distribution of a comprehensive range of cardiovascular risk factors (CVRF) in a Swiss population and assessed whether socioeconomic differences varied by age and gender.

**Methods:**

Participants were 2960 men and 3343 women aged 35–75 years from a population-based survey conducted in Lausanne, Switzerland (CoLaus study). Educational level was the indicator of socioeconomic status used in this study. Analyses were stratified by gender and age group (35–54 years; 55–75 years).

**Results:**

There were large educational differences in the prevalence of CVRF such as current smoking (Δ = absolute difference in prevalence between highest and lowest educational group:15.1%/12.6% in men/women aged 35–54 years), physical inactivity (Δ = 25.3%/22.7% in men/women aged 35–54 years), overweight and obesity (Δ = 14.6%/14.8% in men/women aged 55–75 years for obesity), hypertension (Δ = 16.7%/11.4% in men/women aged 55–75 years), dyslipidemia (Δ = 2.8%/6.2% in men/women aged 35–54 years for high LDL-cholesterol) and diabetes (Δ = 6.0%/2.6% in men/women aged 55–75 years). Educational inequalities in the distribution of CVRF were larger in women than in men for alcohol consumption, obesity, hypertension and dyslipidemia (p<0.05). Relative educational inequalities in CVRF tended to be greater among the younger (35–54 years) than among the older age group (55–75 years), particularly for behavioral CVRF and abdominal obesity among men and for physiological CVRF among women (p<0.05).

**Conclusion:**

Large absolute differences in the prevalence of CVRF according to education categories were observed in this Swiss population. The socioeconomic gradient in CVRF tended to be larger in women and in younger persons.

## Introduction

In high income countries, cardiovascular disease (CVD) disproportionately affects the lower socioeconomic groups [1], probably reflecting an unequal distribution of cardiovascular risk factors (CVRF) across society [2], [3], [4] and differential access to and/or use of treatment [5]. However, the magnitude of socioeconomic inequalities in relation to CVD mortality differs substantially between countries [6], [7]. In Europe, there appears to be a North-South gradient in socioeconomic inequalities in CVD, with larger differences in Northern than in Southern European countries [7].

Between-country variations in the magnitude of socioeconomic inequalities in CVD tend to mirror cross-country differences in the social patterning of CVRF. Indeed, strong socioeconomic inequalities in CVRF have frequently been reported in Northern European regions such as in Scandinavian countries or in the United Kingdom [8], [9], [10], while in several Southern European countries such as Italy, Greece or Spain the association between socioeconomic indicators and CVRF seems to be weaker [11], [12], [13], [14]. For example, Schroder et al. [14] and de Vogli et al. [13] reported a lack of educational/occupational differences in CVRF in Spain and Italy, respectively. Stringhini et al. [15] showed large occupational inequalities in the prevalence of unhealthy behaviors among British civil servants but small inequalities among French employees of the national gas and electricity company. Cavelaars et al. [2] noted a North-South pattern in the social distribution of smoking and vegetable consumption with small associations with educational level in Southern European regions.

These North-South differences might be explained by the fact that CVRF were originally more prevalent in the higher socioeconomic groups and the direction of this association has gradually reversed over the last century [16], [17]. The “social transition” of CVRF from the higher to the lower socioeconomic groups appears to have started earlier in Northern than in Southern Europe, and to have occurred in men before women [18]. In some Southern European countries certain CVRF such as smoking (among women) or low consumption of fruit and vegetables are still more prevalent in the higher socioeconomic groups [11], [19], [20]. For example, Huisman et al. reported large educational differences in current smoking in both Northern and Southern Europe, but in Italy, Spain, Greece and Portugal the socioeconomic gradient in women was inversed, the prevalence of smoking being higher among higher educated women [10]. However, most studies examining the social patterning of CVRF in Southern European countries, including Switzerland, are based on data from the 1990’s [2], [11], [14], [19], [21], [22].

In the French-speaking region of Switzerland, the most recent comprehensive assessment of social inequalities in CVRF dates back to the early 2000s [22]. It showed small but significant socioeconomic differences in the prevalence of several CVRF such as current smoking, physical inactivity, obesity and hypertension (but not hypercholesterolemia) among men. Among women, a similar pattern was observed, but current smoking was not socially patterned. More recent studies examining only one risk factor at a time reported decreasing educational inequalities in smoking [23], but increasing educational differences in overweight and obesity [24].

The overall aim of our study is to provide an updated and comprehensive assessment of social inequalities in major risk factors for lifestyle-related diseases (current smoking, heavy drinking, physical inactivity, overweight and obesity, hypertension, dyslipidemia and diabetes) in a French-speaking Swiss town. As the French-speaking region of Switzerland is generally assimilated to Southern European countries for its CVD profile [25], this study allows assessing whether it is still the case that social inequalities in major CVRF are small in Southern Europe. A key feature of this study is that it additionally examines whether socioeconomic differences in CVRF vary by age and gender.

## Data and Methods

### Study Population and Design

The Colaus study is a cross-sectional population-based study conducted in Lausanne, Switzerland (approximately 180’000 inhabitants). Details of the study have been previously described [26]. Briefly, a simple random sampling of 19,830 participants was drawn, corresponding to 35% of the source population, of which 6738 participants were eventually included. The following inclusion criteria applied: (a) written informed consent; (b) age 35–75 years; (c) willingness to take part in the examination and donate a blood sample; and (d) Caucasian origin. Recruitment began in June 2003 and ended in May 2006. The age and sex distribution of the 6738 participants included in the Colaus study were similar to those of the 19,830 individuals originally sampled. Participants attended the outpatient clinic at the University Hospital of Lausanne (CHUV) in the morning after an overnight fast. Data were collected by trained field interviewers during a single visit lasting about 60 minutes. Venous blood samples were drawn after an overnight fast, and assays were performed by the CHUV Clinical Laboratory on fresh plasma samples within 2 hour of blood collection in a Modular P apparatus (Roche Diagnostics, Switzerland). Information on demographic data, socioeconomic and marital status, lifestyle factors, personal and family history of disease, CVRF and treatment was collected. The study was approved by the Institutional Ethics Committee of the University of Lausanne (Switzerland).

### Measures

#### Socioeconomic status (SES)

*Education* was the indicator of socioeconomic status used in this study. It was assessed as the highest qualification achieved and categorized as “high” (tertiary education), “middle” (upper secondary education or post-secondary non tertiary education, including vocational education) and “low” (lower secondary education or lower) [27].

#### Cardiovascular risk factors (CVRF)

*Current smoking* was assessed using questions on current smoking status and was classified as yes/no. Former smokers were included in the non-smokers category. For current smokers, the *number of pack years* of smoking was calculated by multiplying the number of packs of cigarettes smoked per day (average number of cigarettes smoked per day divided by 20) by the number of years the person reported to have smoked. *Alcohol consumption* was assessed using questions on the number of alcoholic drinks consumed in the past week, then categorized as “abstainers” (0 unit/week), “moderate drinkers” (1–21/1–14 units/week for men/women) or “heavy drinkers” (≥21/≥14 units/week for men/women). We considered both abstaining from alcohol and heavy drinking as CVRF. Participants were classified as *physically active* if they reported participating in a physical activity of more than 20 minutes once a week or more, and as physically inactive otherwise. Body weight and height were measured with participants standing without shoes in light indoor clothing. Body weight was measured in kilograms to the nearest 0.1 kg using a Seca® Scale (Hamburg, Germany), which was calibrated regularly. Height was measured to the nearest 5 mm using a Seca® height gauge (Hamburg, Germany). Waist circumference was measured twice with a non-stretchable tape over the unclothed abdomen at the mid-point between the lowest rib and the iliac crest. The mean of the two measurements was used for analyses [26]. *Body Mass Index* (BMI) was calculated and categorized in three groups (normal <25; overweight 25–29; obese ≥30 kg/m^2^) based on the World Health Organization recommendations [28]. *Abdominal obesity* was considered as a waist circumference ≥102 cm for men and ≥88 cm for women. Blood pressure (BP) was measured three times on the left arm after at least 10 minutes of rest in a seated position using a clinically validated automated oscillometric device (Omron HEM-907, Matsusaka, Japan) with a cuff adapted to the arm circumference. Three readings were obtained and the average of the last two BP readings was used. *Hypertension* was defined as systolic/diastolic BP≥140/90 mmHg or use of antihypertensive medication. *Low HDL-cholesterol* was defined for values <1.0 mmol/l in men and <1.2 mmol/l in women; *high LDL-cholesterol* for a value ≥3.4 mmol/l; *high triglycerides* for a value ≥1.7 mmol/l. *Diabetes* was defined as fasting plasma glucose ≥7.0 mmol/L or glucose lowering treatment.

#### Other covariates

*Place of birth* was classified as “born in Switzerland” or “not born in Switzerland”.

### Statistical Analysis

Statistical analysis was conducted using Stata v.12 (Stata corp, College Station, TX, USA). With the few exceptions mentioned below, all analyses were performed separately for men and women and in two age groups (35–54 years and 55–75 years). We used least squares regression to calculate age and place of birth-adjusted prevalence rates or mean values of CVRF for each educational group. Differences in CVRF prevalence and mean values between the lowest and the highest educational group, with their 95% confidence intervals (CI), were also calculated. As suggested in previous studies [29], [30], relative inequalities in CVRF were examined using the Relative Index of Inequality (RII) calculated by log-binomial regression [31]. The RII is a regression-based index taking into account both the size and relative position of each educational group in the educational hierarchy. To compute the RII, education was transformed into a summary measure ranging from zero (highest level of education) to one (lowest level of education). The population in each educational category was assigned a score corresponding to the midpoint of the relative position of their category in the cumulative population distribution. For example, if the highest educational category comprises 24% of the population, all participants in this category are assigned a value of 0.12 (0.24/2), and if the second category comprises 30% of the population, the corresponding value is 0.27 (0.12+ [0.3/2]), and so forth. The RII was calculated using log-binomial regression, as the RII by logistic regression has been shown to produce biased estimates of relative inequalities when the prevalence of the health outcome is relatively high (i.e.: >10%) [30]. As such, the RII can be interpreted as the prevalence ratio between the two ends of the educational hierarchy [30]. Log-binomial regressions were adjusted for age (treated as a continuous variable) and place of birth. Analyses including HDL-cholesterol were additionally adjusted for oral contraceptive intake among women. In order to test whether the associations between education and CVRF differed by gender or by age, interaction terms between education (lowest versus highest education in analysis of absolute inequalities and RII in analysis of relative inequalities) and sex or between education and age group were included in the different regression models described.

## Results

From the initial 6738 participants, 435 (6% of the original sample) were excluded because of missing values on one or more covariates (N = 18 for education, N = 157 for alcohol consumption, N = 114 for physical inactivity and N <20 for the other CVRF, categories not mutually exclusive). Hence, 6303 participants (53% women) were included in the present analyses. Excluded women were slightly older than those included in the study (p = 0.03), but there were no age differences between included and excluded men. Excluded participants were more likely to have CVRF than those included in the analysis (for example, OR = 1.54; 95%CI: 1.25; 1.90 for smoking, OR = 1.29; 95%CI: 1.00; 1.66 for obesity and OR = 2.14; 95%CI: 1.55; 2.94) and they were also more likely to be in the lowest educational group than those included in the study (OR = 1.69; 95%CI: 1.27; 2.27). However, educational inequalities in CVRF were similar in both the excluded and included samples (p for interaction between education and inclusion status>0.05 for smoking, obesity or diabetes).

**Table 1** shows the characteristics of the participants included in the study. Mean age was 52 years for both men and women. One half of participants reported “lower than secondary” education (52.8% of men and 57.8% of women). The distribution of participants across educational categories was similar in the two age groups for men, while women in the older age group tended to report a lower educational level than those in the younger group. The majority of men and women were born in Switzerland.

**Table 1:** Socio-demographic characteristics by gender and age group.

	MEN			WOMEN		
	Overall	35–54 years	55–75 years	Overall	35–54 years	55–75 years
**N (%)**	2960 (47.0)	1727 (58.3)	1233 (41.7)	3343 (53.0)	1842 (55.1)	1501 (44.9)
**Age (mean, SD)**	52.2 (10.8)	44.3 (5.3)	63.1 (5.8)	52.9 (10.7)	44.6 (5.4)	63.0 (5.7)
**Education, N (%)**						
Tertiary	710 (24.0)	463 (26.8)	247 (20.0)	550 (16.5)	396 (21.5)	154 (10.3)
Post secondary/secondary	685 (23.2)	391 (22.6)	294 (23.8)	859 (25.7)	504 (27.4)	355 (23.7)
Lower than secondary	1565 (52.8)	873 (50.6)	692 (56.2)	1934 (57.8)	942 (51.1)	992 (66.0)
**Born in Switzerland, N (%)**						
Yes	1765 (59.6)	910 (52.7)	855 (69.3)	2035 (60.9)	1016 (55.2)	1019 (67.9)
No	1195 (40.4)	817 (47.3)	378 (30.7)	1308 (39.1)	826 (44.8)	482 (32.1)

SD: standard deviation.

### Absolute Inequalities in CVRF

For men, age and place of birth-adjusted prevalence and mean values of CVRF by educational level and age group are presented in **Table 2**. Lower education was associated with higher levels of CVRF with a marked dose-response pattern (p for linear trends <0.05 for all CVRF apart from LDL-cholesterol in the younger age group and alcohol consumption, LDL and HDL-cholesterol in the older age group). There was a 15% (95%CI: 10.0; 20.2) difference in the prevalence of smoking between the lowest and the highest educational group in the youngest age group, but there were no significant educational differences in smoking prevalence in the oldest age group [Δ = 3.1% (95%CI: −3.0; 9.2)]. In both age groups, the number of pack-years smoked increased with decreasing educational level. Physical inactivity, overweight, obesity and abdominal obesity were also far more prevalent in the lowest as compared with the highest educational group (Δ = 25.3%/19.4% in the youngest/oldest age group for physical inactivity; 14.7%/12.5% for overweight; 8.6%/14.2% for obesity and 9.3%/4.6% for abdominal obesity). Large differences were also seen for hypertension (particularly in the oldest age group (Δ = 16.7%)), but less so for dyslipidemia and diabetes. Absolute educational differences in CVRF tended to be larger in the younger than in the older age group for smoking, heavy drinking, physical inactivity and abdominal obesity, but they were larger in the older age group for obesity and hypertension.

**Table 2:** Age-adjusted prevalence and mean values of selected cardiovascular risk factors by level of education and age group among men (N = 2960).

		Age group 35–54 years (N = 1727)						Age group 55–75 years (N = 1233)						
		Educational level						Educational level						
	Overall	High	Mid	Low	*p* a	*Δ* b	(95% CI)	High	Mid	Low	*p* a	*Δ* b	(95% CI)	*P* c
**Health behaviors**														
Current smoking (%)	**29.4**	21.6	29.1	36.7	*0.000*	15.1	(10.0; 20.2)	22.7	24.3	25.8	*0.314*	3.1	(−3.0; 9.2)	***0.004***
Pack-years (mean)^d^	**24.2**	13.7	18.8	23.0	*0.000*	9.2	(5.7; 12.7)	19.2	25.8	34.0	*0.001*	15.0	(6.6;26.5)	***0.341***
No alcohol consumption (%)	**18.0**	16.1	19.1	22.1	*0.008*	6.0	(1.6; 10.4)	13.7	14.3	14.9	*0.650*	1.2	(−3.8; 6.1)	***0.117***
Heavy drinking (%)	**12.2**	6.1	8.9	11.8	*0.001*	5.7	(2.4; 9.0)	15.8	15.1	14.4	*0.575*	−1.4	(−6.5; 3.6)	***0.009***
Physical inactivity (%)	**36.6**	20.2	32.8	45.5	*0.000*	25.3	(20.2; 30.5)	21.9	31.6	41.3	*0.000*	19.4	(12.8; 26.1)	***0.111***
**Body mass index**(mean, kg/m^2^)	**26.6**	25.0	25.7	26.3	*0.000*	1.3	(0.9; 1.8)	26.4	27.2	27.9	*0.000*	1.4	(0.9; 2.0)	***0.827***
Overweight e (%)	**62.0**	45.2	52.6	59.9	*0.000*	14.7	(9.3; 20.2)	62.4	68.6	74.9	*0.000*	12.5	(6.1; 18.9)	***0.546*** ***546***
Obesity e (%)	**17.1**	6.4	10.7	15.1	*0.000*	8.6	(5.1; 12.2)	12.8	19.9	27.0	*0.000*	14.2	(8.3; 20.1)	***0.126***
**Waist circumference**(mean, cm)	**95.6**	91.0	92.3	93.6	*0.000*	2.6	(1.5; 3.8)	97.6	98.7	99.8	*0.005*	2.3	(0.7; 3.8)	***0.608***
Abdominal obesity e (%)	**26.6**	10.8	15.5	20.2	*0.000*	9.3	(5.2; 13.4)	34.8	37.1	39.4	*0.186*	4.6	(−2.2; 11.5)	***0.220***
**Blood pressure** (mmHg)														
Systolic (mean)	**131.8**	124.7	126.1	127.5	*0.000*	2.9	(1.4; 4.3)	135.3	137.8	140.2	*0.000*	4.9	(2.4; 7.3)	***0.106***
Diastolic (mean)	**81.2**	78.8	79.8	80.8	*0.001*	2.0	(0.8; 3.1)	81	82.1	83.3	*0.003*	2.3	(0.8; 3.9)	***0.710***
Hypertension e (%)	**37.2**	18.3	21.9	25.4	*0.002*	7.1	(2.5; 11.7)	43.5	51.8	60.2	*0.000*	16.7	(9.8; 23.6)	***0.023***
**HDL-cholesterol**(mean, mmol/l)	**1.44**	1.45	1.43	1.41	*0.099*	−0.03	(−0.07; 0.01)	1.49	1.48	1.46	*0.176*	−0.04	(−0.09; 0.02)	***0.826***
Low HDL-cholesterol e (%)	**4.2**	3.1	4.5	5.9	*0.020*	2.8	(0.4; 5.2)	2.8	3.1	3.3	*0.650*	0.6	(−1.9; 3.1)	***0.182***
**LDL-cholesterol**(mean, mmol/l)	**3.39**	3.36	3.39	3.42	*0.250*	0.06	(−0.04; 0.16)	3.43	3.41	3.39	*0.472*	−0.05	(−0.17; 0.08)	***0.008***
High LDL-cholesterol e (%)	**49.9**	47.5	49.4	51.4	*0.162*	4.0	(−1.6; 9.6)	49.9	50.8	51.7	*0.623*	1.8	(−5.3; 8.8)	***0.366***
**Triglycerides** (mean, mmol/l)	**1.48**	1.37	1.43	1.50	*0.005*	0.12	(0.04; 0.22)	1.38	1.48	1.57	*0.001*	0.18	(0.08; 0.29)	***0.769***
High triglycerides e (%)	**32.5**	26.4	29.5	32.7	*0.015*	6.4	(1.2; 11.5)	28.7	32.6	36.5	*0.023*	7.8	(1.1; 14.5)	***0.994***
**Fasting glucose**(mean, mmol/l)	**5.76**	5.45	5.54	5.63	*0.001*	0.18	(0.08; 0.29)	5.89	5.96	6.03	*0.132*	0.15	(−0.04; 0.34)	***0.543***
Diabetes e (%)	**8.9**	1.5	3.1	4.7	*0.003*	3.1	(1.1; 5.2)	11.1	14.1	17.1	*0.021*	6.0	(0.9; 11.0)	***0.253***

BP: blood pressure; CI: confidence interval; HDL: high-density lipoprotein; LDL: low-density lipoprotein. Prevalence and mean values are adjusted for age and place of birth (Switzerland or outside Switzerland).

a *p* for linear trend across socioeconomic categories.

b Difference in prevalence/mean between the highest and the lowest educational category.

c *p* for interaction between educational level and age group.

d Analyses restricted to current smokers (N = 515 in the 35–54 years group and N = 293 in the 55–75 years group). 32 smokers with missing information on pack-years were not included.

e Overweight: BMI ≥25 kg/m^2^ and <30 kg/m^2^; obesity: BMI ≥30 kg/m^2^; abdominal obesity: waist circumference ≥102/88cm in men/women; hypertension: BP≥140/90 mmHg or taking BP treatment; low HDL-cholesterol: <1.0/1.2 mmol/l in men/women; high LDL-cholesterol: ≥3.4 mmol/l; high triglycerides: ≥1.7 mmol/l; diabetes:fasting glucose ≥7.0 mmol/l or taking diabetes treatment.

For women, the prevalence and mean values of CVRF according to educational level and age group are presented in **Table 3**. As for men, most CVRF showed a linear association with educational level (p for linear trends <0.05 for all CVRF apart from heavy drinking in the younger age group and smoking, diastolic blood pressure, HDL and LDL-cholesterol and diabetes in the older age group). In the younger age group, but not in the older, large absolute inequalities were observed for current smoking (Δ = 12.6%). Physical inactivity (Δ = 22.7%/21.2% in the younger/older age group), overweight (Δ = 22.9%/27.9%), obesity (Δ = 10.3%/14.8%), abdominal obesity (Δ = 15.7%/21.6%), hypertension (Δ = 8.6%/11.4%) and dyslipidemia (Δ = 9.5%/7.8% for high LDL-cholesterol) were more prevalent in the lowest educational group in both age groups.

**Table 3:** Age-adjusted prevalence and mean values of selected cardiovascular risk factors by level of education and age group among women (N = 3343).

		Age group 35–54 years (N = 1842)						Age group 55–75 years (N = 1501)						
		Educational level						Educational level						
	Overall	High	Mid	Low	*p* a	*Δ* b	(95% CI)	High	Mid	Low	*p* a	*Δ* b	(95% CI)	*P* c
**Health behaviors**														
Current smoking (%)	**23.6**	20.4	26.7	33	*0.000*	12.6	(7.5; 17.8)	20.3	19.9	19.6	*0.813*	−0.7	(−6.9; 5.4)	***0.000*** **.** ***00***
Pack-years (mean)^d^	**20.9**	12.5	16.9	19.2	*0.000*	6.3	(2.9; 9.7)	16.7	23.8	28.7	*0.002*	11.4	(4.1; 18.7)	***0.180***
No alcohol consumption (%)	**38.0**	26.1	35.9	45.7	*0.000*	19.6	(14.2; 25.1)	30.2	35.3	40.4	*0.007*	10.3	(2.8; 17.7)	***0.258***
Heavy drinking (%)	**4.7**	4.5	4.5	4.6	*0.894*	0.2	(−2.2; 2.6)	6.4	5.3	4.2	*0.187*	−2.2	(−5.5; 1.1)	***0.115***
Physical inactivity (%)	**34.8**	20.3	31.7	43.0	*0.000*	22.7	(17.4; 28.0)	18.8	29.4	40	*0.000*	21.2	(14.0; 28.5)	***0.776***
**Body mass index**(mean, kg/m^2^)	**25.0**	23.0	24.1	25.2	*0.000*	2.2	(1.7; 2.8)	23.6	25.1	26.6	*0.000*	3.0	(2.3; 3.8)	***0.086***
Overweight e (%)	**41.8**	21.3	32.7	44.1	*0.000*	22.9	(17.5; 28.2)	28.5	42.4	56.4	*0.000*	27.9	(20.3; 35.5)	***0.305***
Obesity e (%)	**13.8**	5.0	10.1	15.2	*0.000*	10.3	(6.6; 13.9)	5.4	12.8	20.3	*0.000*	14.8	(9.1; 20.6)	***0.187***
**Waist circumference**(mean, cm)	**83.0**	77.5	79.9	82.3	*0.000*	4.8	(3.5; 6.1)	81.1	84.6	88	*0.000*	7.0	(5.1; 8.9)	***0.061***
Abdominal obesity e (%)	**31.7**	13.4	21.3	29.1	*0.000*	15.7	(10.9; 20.5)	26.4	37.2	47.9	*0.000*	21.6	(14; 29.1)	***0.139***
**Blood pressure** (mmHg)														
Systolic (mean)	**124.0**	115.2	116.9	118.7	*0.000*	3.5	(1.9; 5.1)	128.8	131.2	133.6	*0.001*	4.8	(2.1; 7.6)	***0.241***
Diastolic (mean)	**77.4**	74.6	75.8	77.0	*0.000*	2.4	(1.2; 3.6)	78.0	78.7	79.4	*0.101*	1.4	(−0.3; 3.0)	***0.087***
Hypertension e (%)	**25.0**	8.0	12.3	16.6	*0.000*	8.6	(4.8; 12.5)	31.8	37.5	43.2	*0.003*	11.4	(4.0; 18.8)	***0.369***
**HDL-cholesterol** f(mean, mmol/l)	**1.81**	1.89	1.81	1.73	*0.000*	−0.16	(−0.21; −0.11)	1.93	1.86	1.79	*0.000*	−0.14	(−0.21; −0.07)	***0.970***
Low HDL-cholesterol e (%)	**4.3**	0.6	3.7	6.8	*0.000*	6.2	(3.8; 8.6)	2.7	3.8	4.8	*0.199*	2.1	(−1.1; 5.2)	***0.027***
**LDL-cholesterol**(mean, mmol/l)	**3.26**	2.89	2.99	3.10	*0.000*	0.21	(0.12; 0.31)	3.50	3.55	3.59	*0.172*	0.09	(−0.04; 0.24)	***0.036***
High LDL-cholesterol e (%)	**42.2**	24.1	28.8	33.6	*0.000*	9.5	(4.4; 14.7)	52.3	56.2	60.1	*0.044*	7.8	(0.2; 15.4)	***0.330***
**Triglycerides**(mean, mmol/l)	**1.12**	1.37	1.43	1.50	*0.005*	0.13	(0.04; 0.22)	1.38	1.48	1.57	*0.001*	0.18	(0.08; 0.29)	***0.647***
High triglycerides e (%)	**15.0**	5.6	9.7	13.8	*0.000*	8.2	(4.6; 11.7)	14.9	18.7	22.4	*0.018*	7.5	(1.3; 13.8)	***0.859***
**Fasting glucose**(mean, mmol/l)	**5.32**	5.11	5.16	5.21	*0.018*	0.09	(0.02; 0.17)	5.33	5.45	5.58	*0.008*	0.25	(0.07; 0.43)	***0.165***
Diabetes e (%)	**3.2**	0.4	1.1	1.7	*0.049*	1.3	(0.0; 2.6)	4.1	5.4	6.7	*0.168*	2.6	(−1.1; 6.3)	***0.414***

BP: blood pressure; CI: confidence interval; HDL: high-density lipoprotein; LDL: low-density lipoprotein. Prevalence and mean values are adjusted for age and place of birth (Switzerland or outside Switzerland).

a *p* for linear trend across socioeconomic categories.

b Difference in prevalence/mean between the highest and the lowest educational category.

c *p* for interaction between educational level and age group.

d Analyses restricted to current smokers (N = 503 in the 35–54 years group and N = 288 in the 55–75 years group). 31 smokers with missing information on pack-years were not included.

e Overweight: BMI ≥25 kg/m^2^ and <30 kg/m^2^; obesity: BMI ≥30 kg/m^2^; abdominal obesity: waist circumference ≥102/88cm in men/women; hypertension: BP≥140/90 mmHg or taking BP treatment; low HDL-cholesterol: <1.0/1.2 mmol/l in men/women; high LDL-cholesterol: ≥3.4 mmol/l; high triglycerides: ≥1.7 mmol/l; diabetes: fasting glucose ≥7.0 mmol/l or taking diabetes treatment.

f Analyses for HDL-cholesterol are additionally adjusted for oral contraceptive use.

### Relative Inequalities in CVRF

Results for relative educational inequalities in CVRF are shown in **Table 4**. Participants at the bottom end of the educational hierarchy were more likely to be current smokers than those at the top, but in analysis stratified by age group the association of smoking status with education was evident only in the younger age group (p for interaction between education and age group<0.05). In general, relative educational inequalities in CVRF were larger in the younger age group (although interaction terms reached statistical significance only for smoking, heavy drinking and abdominal obesity in men and for smoking, hypertension, and dyslipidemia in women). For example, men at the bottom of the educational hierarchy were more than four times more likely to have diabetes than those at the top in the younger age group [RII = 4.61 (95%CI:1.62; 13.10)], but only 1.9 times more likely in the older age group [RII = 1.89 (95%CI:1.10; 3.27)]. The corresponding figure for women was RII = 5.12 (95%CI:0.84; 31.33) among those 35–54 years and RII = 1.87 (95%CI:0.71; 4.88) among those 55–75 years. Among women, relative inequalities were particularly strong for obesity [RII = 4.77 (95%CI:3.15; 7.22)] and low HDL-cholesterol [RII = 5.62 (95%CI:2.64; 12.94)]. Educational inequalities in alcohol abstinence, hypertension and dyslipidemia in the younger age group and in abdominal obesity in the older age group were larger in women than in men (all p<0.05).

**Table 4:** Relative educational inequalities in selected cardiovascular risk factors by gender and age group.

	MEN				WOMEN				GENDER DIFFERENCES			
	Overall (N = 2963)	35–54 y (N = 1727)	55–75 y (N = 1233)		Overall (N = 3343)	35–54 y (N = 1842)	55–75 y (N = 1501)		Overall	35–54y	55–75y	
	RII (95%CI)^a^	RII (95%CI)^a^	RII (95%CI)^a^	*P* c	RII (95%CI)^ ab^	RII (95%CI)^ ab^	RII (95%CI)^ ab^	*P* c	*P* d	*P* d	*P* d	
**Behavioral risk factors**												
Smoking	1.85 (1.48;2.31)	2.26 (1.71;2.98)	1.22 (0.83;1.79)	*0.011*	1.55 (1.22;1.98)	1.93 (1.44;2.58)	0.93 (0.6;1.45)	*0.001*	***0.365***	***0.774***	***0.287***	
No alcohol consumption	1.44 (1.07;1.94)	1.63 (1.13;2.34)	1.12 (0.66;1.88)	*0.163*	2.03 (1.69;2.45)	2.29 (1.82;2.87)	1.52 (1.12;2.06)	*0.171*	***0.027***	***0.120***	***0.160***	
Heavy drinking	1.46 (0.99;2.14)	2.75 (1.55;4.88)	0.88 (0.53;1.47)	*0.002*	0.75 (0.40;1.40)	1.09 (0.48;2.46)	0.49 (0.19;1.26)	*0.087*	***0.066***	***0.086***	***0.333***	
Physical inactivity	2.98 (2.43;3.65)	3.27 (2.52;4.25)	2.53 (1.83;3.51)	*0.141*	2.84 (2.31;3.50)	2.93 (2.26;3.79)	2.64 (1.87;3.73)	*0.639*	***0.707***	***0.539***	***0.902***	
Obesity^e^	2.95 (2.09;4.16)	3.30 (1.95;5.58)	2.82 (1.80;4.43)	*0.531*	4.77 (3.15;7.22)	4.74 (2.72;8.26)	4.76 (2.56;8.86)	*0.991*	***0.177***	***0.347***	***0.215***	
Abdominal obesity^e^	1.48 (1.17;1.87)	2.42 (1.60;3.66)	1.18 (0.89;1.56)	*0.003*	2.60 (2.06;3.27)	3.11 (2.20;4.41)	2.24 (1.65;3.04)	*0.153*	***0.012***	***0.380***	***0.005***	
**Physiological risk factors**												
Hypertension^d^	1.50 (1.26;1.79)	1.64 (1.19;2.26)	1.55 (1.26;1.91)	*0.512*	1.85 (1.43;2.39)	2.84 (1.75;4.61)	1.57 (1.16;2.11)	*0.013*	***0.017***	***0.048***	***0.706***	
Low HDL-cholesterol^e^	2.06 (1.04;4.09)	2.45 (1.08;5.58)	1.32 (0.39;4.48)	*0.358*	5.62 (2.64;11.94)	12.67 (4.48;35.85)	1.79 (0.59;5.42)	*0.008*	***0.048***	***0.019***	***0.763***	
High LDL-cholesterol^e^	1.11 (0.97;1.27)	1.16 (0.97;1.38)	1.06 (0.86;1.31)	*0.291*	1.37 (1.16;1.62)	1.75 (1.33;2.30)	1.21 (0.99;1.48)	*0.004*	***<0.001***	***0.021***	***0.183***	
High triglycerides^e^	1.43 (1.17;1.76)	1.40 (1.07;1.83)	1.50 (1.10;2.05)	*0.990*	2.45 (1.69;3.54)	3.56 (2.03;6.23)	1.88 (1.15;3.08)	*0.041*	***<0.001***	***0.001***	***0.369***	
Diabetes^e^	2.20 (1.35;3.58)	4.61 (1.62;13.1)	1.89 (1.09;3.27)	*0.097*	2.35 (1.00;5.53)	5.12 (0.84;31.33)	1.87 (0.71;4.88)	*0.308*	***0.878***	***0.948***	***0.988***	

CI: confidence interval; HDL: high-density lipoprotein; LDL: low-density lipoprotein; RII: relative index of inequality; y:years.

a Adjusted for age and place of birth (Switzerland or outside Switzerland).

b Analyses for HDL-cholesterol are additionally adjusted for oral contraceptive use.

c *p* for interaction between RII and age group.

d *p* for interaction between RII and sex.

e Obesity: BMI ≥30 kg/m^2^; abdominal obesity: waist circumference ≥102/88cm in men/women; hypertension: BP≥140/90 mmHg or taking BP treatment; low HDL-cholesterol: <1.0/1.2 mmol/l in men/women; high LDL-cholesterol: ≥3.4 mmol/l; high triglycerides: ≥1.7 mmol/l; diabetes: fasting glucose ≥7.0 mmol/l or taking diabetes treatment.

### Sensitivity Analyses

About 40% of participants were not born in Switzerland. As education can have different meanings in different populations, depending on the school system and the level of economic development, we repeated the analyses for relative inequalities in CVRF stratifying for place of birth (Switzerland or not Switzerland). In general, there were no substantial differences in educational inequalities in CVRF by place of birth **(Table S1).** However, inequalities in heavy drinking and diabetes were larger among men not born in Switzerland, and inequalities in obesity were larger among women born in Switzerland (all p<0.05). About 6% of participants had missing values on one or more covariates. As missingness was found to be patterned by education, we assessed whether missing data could have biased our results. Analyses for relative educational inequalities in CVRF were rerun using multiple multivariate imputation (STATA procedures “ice/micombine”) to replace missing values. Results did not differ from those reported in the main analysis. Although socioeconomic status is a complex concept, we focused on educational level in this study. However, analyses were also performed using occupational position as the indicator of SES for the 4512 participants who were currently working. Overall, results were very similar to those using education as an indicator of SES. However, in general socioeconomic differences in CVRF tended to be more pronounced for education than for occupational position, especially among women. Finally, we repeated all analyses adjusting for marital status and results were virtually unchanged. All results from sensitivity analysis not shown in Table S1 are available upon request.

## Discussion

We found large absolute differences in the prevalence of CVRF according to educational level in this Swiss population. Moreover, relative inequalities by education differed by gender and tended to be greater in the younger than in the older age group, particularly for behavioral risk factors among men and for physiological risk factors among women.

### Overall Prevalence of CVRF

Prevalence estimates of smoking, physical inactivity, obesity, and hypertension in our study were comparable to other population-based estimates (several of them telephone health surveys) of the Swiss general population [23], [32], [33], [34]. On the other hand, the prevalence of measured hypercholesterolemia and diabetes in Colaus was higher than self-reported prevalence from the Swiss health surveys [35], [36], or than that measured in the neighboring region of Geneva [22], [37]. The prevalence of overweight and obesity, hypertension, high triglycerides and diabetes was higher in men than in women, as reported previously in Switzerland [38].

### Absolute Educational Differences in CVRF

Overall, the prevalence of CVRF was lower in higher socioeconomic groups, consistent with general findings in high income countries [2], [3], [4]. Absolute socioeconomic differences were particularly large for behaviors such as smoking and physical activity, and anthropometric measures such as weight (reflecting the balance between physical activity and diet). This was particularly true among women. This result is in line with findings from recent studies reporting strong educational inequalities in physical inactivity and obesity in Switzerland [32], [39]. Absolute educational differences were also large for hypertension but smaller for dyslipidemia and diabetes, as observed previously [40].

### Relative Educational Differences in CVRF

Relative educational inequalities differed by age and gender for several CVRF. Among both men and women, the educational gradient in current smoking was stronger in the younger than in the older age group. It has been observed that the smoking epidemic initially spread in the high socioeconomic groups, later reached the lower socioeconomic groups, and started declining first in the high socioeconomic group [18], [21]. In addition, the “social transition” of smoking usually starts earlier in men than in women, and in Europe it was delayed in Southern Europe as compared with Northern Europe [18]. In a study conducted in Geneva (Switzerland) in the 1990s, smoking was still more prevalent among the higher educated women [19], but there were no educational differences in current smoking among young participants (35–44 years in 1993–95). The current study suggests that the social transition in smoking is now completed in Switzerland.

Gender and age differences were also observed for the association between education and heavy drinking. Low-educated young men were more likely to report heavy drinking than young men with high education, but the inverse was observed among older men. Among women, heavy drinking tended to be more common in the higher educational group, as observed previously in Switzerland and other European countries [39], [41], but the associations were not statistically significant. Relative inequalities in physical inactivity were very large but did not differ by age and gender. Conversely, the social patterning of obesity was stronger in women, as previously reported [39], [42]. This was mostly due to the very low prevalence of obesity among the highly educated women. It has been hypothesized that this might reflect a stronger social pressure for thinness on women with a high socioeconomic status than on women with a low socioeconomic status, in addition to greater health consciousness [43].

Hypertension was also strongly socially patterned, as reported previously in Switzerland [22]. Although absolute inequalities in dyslipidemia and diabetes were not large, relative inequalities were strong in Lausanne compared with other countries [44]. For example, young women with a low educational level were more than 10 times more likely to have low HDL-cholesterol and 5 times more likely to have diabetes than their more advantaged counterparts. This might be related to the observed inequalities in physical inactivity and obesity among younger women.

For most CVRF and for both genders, relative educational inequalities were stronger in the younger (35–54 years) than in the older (55–75 years) age group. This could either mirror cohort differences in the social patterning of CVRF, with greater social inequalities in younger cohorts, or reflect a decrease in social inequalities in CVRF with ageing. As reported earlier, several studies conducted in Southern Europe (including one study in the French-speaking part of Switzerland) -mostly based on data from the early 1990s- found a small or null socioeconomic gradient in CVRF [2], [11], [13], [14], [19], [21], [22]. Our study is one of the first conducted in a Southern European country to find large socioeconomic differences in CVRF, which may hint at either a new situation in Southern Europe or at a difference between Switzerland and other Southern European countries. If the first hypothesis is true, the fact that inequalities in CVRF tended to be stronger among younger than older participants may translate into an increase in social inequalities in adverse CVD outcomes over the next decades. Alternatively, smaller inequalities in CVRF in the older age group could be explained by the fact that relative inequalities in CVRF might decline with age as a result of increasing prevalence of adverse CVRF across socioeconomic groups or because of selection effects. However, both explanations remain speculative as the cross sectional nature of the study precludes distinguishing between age and cohort effects.

Evidence for a prominent role of behavioral and biological risk factors such as those examined in this paper in explaining social inequalities in cardiovascular disease incidence and mortality is accumulating [45], [46], [47]. The determinants of the uneven distribution of CVRF across socioeconomic groups remain poorly understood, but likely include socioeconomic differences in several domains such as social norms, physical living and working environments, health education, health consciousness, attitude and motivation, psycho-social characteristics, and access to and utilization of health care [48], [49], [50]. We could not examine the role of this broader context in relation to our findings, as these factors were not assessed in our study. Further studies will be needed to elucidate the relative importance of specific factors in the social patterning of CVRF if effective policies to reduce social inequalities in health are to be implemented.

### Strengths and Limitations

The main strength of this study was the availability of a large number of CVRF in a population-based survey covering a wide age range. This study also has potential limitations. The first relates to the inability of the cross-sectional design to distinguish between cohort and age effects. While we speculate that cohort-related changes might be taking place in the social patterning of CVRF, consistently with data from cohort studies or from repeated cross sectional surveys in other populations, we cannot exclude that the observed cohort differences in our study are accountable by age-related changes in behaviors. Second, measurement of socioeconomic position is challenging. Education is a valid indicator of SES as it allows for comparison of men and women and is applicable to the non-working population. However, it can have a different meaning for different birth cohorts, due to secular trends in educational attainment across generations [51]. Our sensitivity analysis using occupational position showed that our findings hold across indicators of socioeconomic status. Finally, health behaviors (smoking, alcohol consumption and physical activity) were self-reported and it has been shown that questionnaire-based measures are not entirely reliable [52], [53].

### Conclusions

This study shows that large socioeconomic differences exist in the prevalence of several CVRF in a country enjoying one of the highest life expectancies at birth and one of the highest gross domestic products per capita in the world [54]. Although the overall prevalence of several CVRF was higher in men than in women, social inequalities tended to be greater in women. Socioeconomic gradients in CVRF were larger in the younger than in the older generations, suggesting that social inequalities in CVD might widen over the next decades. Further research is needed in order to elucidate the mechanisms underlying social inequalities in CVRF.

## Supporting Information

Table S1 **Relative educational inequalities in cardiovascular risk factors by gender and place of birth.**
(DOCX)
