# Supplementary material for: Age and Gender Differences in the Social Patterning of Cardiovascular Risk Factors in Switzerland: The CoLaus Study
Source: PLoS One. 2012 Nov 13;7(11):e49443. doi: 10.1371/journal.pone.0049443 (PMC3496703; doi:10.1371/journal.pone.0049443)
Supplement: Table S1 — Relative educational inequalities in cardiovascular risk factors by gender and place of birth. (DOCX) [file pone.0049443.s001.docx]

TABLE S1. Relative educational inequalities in cardiovascular risk factors by gender and place of birth

|  | **MEN** | | | **WOMEN** | | |
| --- | --- | --- | --- | --- | --- | --- |
|  | **Born in CH (N=1765)** | **Not born in CH (N=1195)** |  | **Born in CH (N=2035)** | **Not born in CH (N=1308)** |  |
|  |  |  |  |  |  |  |
|  | **RII (95%CI)^a^** | **RII (95%CI)^a^** | ***P^b^*** | **RII (95%CI)^a^** | **RII (95%CI)^a^** | ***P^b^*** |
| Smoking | 1.59 (1.19;2.12) | 2.25 (1.58;3.19) | *0.189* | 1.95 (1.41;2.70) | 1.33 (0.91;1.93) | *0.163* |
| No alcohol consumption | 1.94 (1.25;3.01) | 1.06 (0.71;1.59) | *0.052* | 1.83 (1.37;2.43) | 2.14 (1.68;2.72) | *0.853* |
| Heavy drinking | 0.96 (0.59;1.56) | 2.75 (1.44;5.23) | *0.014* | 1.08 (0.50;2.35) | 0.38 (0.12;1.14) | *0.290* |
| Physical inactivity | 2.97 (2.20;4.01) | 2.91 (2.20;3.86) | *0.836* | 3.53 (2.50;4.98) | 2.47 (1.91;3.21) | *0.075* |
| Obesity **^c^** | 2.77 (1.78;4.31) | 3.11 (1.81;5.36) | *0.698* | 8.16 (4.23;15.77) | 3.06 (1.77;5.27) | *0.027* |
| Abdominal obesity **^c^** | 1.58 (1.18;2.12) | 1.29 (0.87;1.92) | *0.506* | 2.70 (1.95;3.74) | 2.49 (1.78;3.47) | *0.773* |
| Hypertension **^c^** | 1.52 (1.22;1.90) | 1.46 (1.10;1.96) | *0.941* | 2.29 (1.61;3.24) | 1.38 (0.94;2.03) | *0.222* |
| Low HDL-cholesterol **^c^** | 2.28 (0.89;5.86) | 1.75 (0.65;4.71) | *0.668* | 6.79 (2.26;20.44) | 5.05 (1.78;14.28) | *0.889* |
| High LDL-cholesterol **^c^** | 1.12 (0.93;1.33) | 1.09 (0.88;1.35) | *0.812* | 1.40 (1.12;1.75) | 1.34 (1.04;1.72) | *0.922* |
| High triglycerides **^c^** | 1.45 (1.11;1.89) | 1.32 (0.95;1.83) | *0.616* | 2.83 (1.73;4.61) | 2.00 (1.12;3.58) | *0.449* |
| Diabetes **^c^** | 1.47 (0.82;2.63) | 4.89 (1.98;12.08) | *0.025* | 2.91 (0.88;9.64) | 1.87 (0.55;6.37) | *0.675* |

CI: confidence interval; CH: Switzerland; HDL: high-density lipoprotein; LDL: low-density lipoprotein; RII: relative index of inequality.

^a^ Adjusted for age.

^b^ *p* for interaction between RII and place of birth.

^c^ Obesity: BMI ≥30 kg/m^2^; abdominal obesity: a waist circumference ≥ 102/88 cm in men/women; hypertension: BP ≥140/90 mmHg or taking BP treatment; low HDL-cholesterol: <1.0/1.2 mmol/l in men/women; high LDL-cholesterol: ≥ 3.4 mmol/l; high triglycerides: ≥ 1.7 mmol/l; diabetes: fasting glucose ≥ 7.0 mmol/l or taking diabetes treatment.
